# Gremlin-1 Promotes Metastasis of Breast Cancer Cells by Activating STAT3-MMP13 Signaling Pathway

**DOI:** 10.3390/ijms21239227

**Published:** 2020-12-03

**Authors:** Nam Ji Sung, Na Hui Kim, Young-Joon Surh, Sin-Aye Park

**Affiliations:** 1 Department of ICT Environmental Health System, Graduate School, Soonchunhyang University, Asan-si 31538, Korea; nam19302@naver.com (N.J.S.); buils457@naver.com (N.H.K.); 2 Tumor Microenvironment Global Core Research Center, College of Pharmacy, Seoul National University, Seoul 08826, Korea; surh@snu.ac.kr; 3 Department of Biomedical Laboratory Science, College of Medical Sciences, Soonchunhyang University, Asan-si 31538, Korea

**Keywords:** Gremlin-1, breast cancer, lung metastasis, MMP13, STAT3

## Abstract

Gremlin-1 (GREM1), one of the bone morphogenetic protein (BMP) antagonists, can directly bind to BMPs. GREM1 is involved in organogenesis, tissue differentiation, and organ fibrosis. Recently, numerous studies have reported the oncogenic role of GREM1 in cancer. However, the role of GREM1 in metastasis of breast cancer cells and its underlying mechanisms remain poorly understood. The role of GREM1 in breast cancer progression was assessed by measuring growth, migration, and invasion of breast cancer cells. An orthotopic breast cancer mouse model was used to investigate the role of GREM1 in lung metastasis of breast cancer cells. *GREM1* knockdown suppressed the proliferation of breast cancer cells, while its overexpression increased their growth, migration, and invasion. Cells with *Grem1*-knockdown showed much lower tumor growth rates and lung metastasis than control cells. GREM1 enhanced the expression of matrix metalloproteinase 13 (MMP13). A positive correlation between *GREM1* and *MMP13* expression was observed in breast cancer patients. GREM1 activated signal transducer and activator of transcription 3 (STAT3) transcription factor involved in the expression of MMP13. Our study suggests that GREM1 can promote lung metastasis of breast cancer cells through the STAT3-MMP13 pathway. In addition, GREM1 might be a promising therapeutic target for breast cancer metastasis.

## 1. Introduction

Breast cancer is still one of the most common cancers in women around the world, although there have been many advances in its diagnosis and treatment in the past decades [1]. Breast cancer is known to be highly treatable if detected early, but is one of the cancers with a high risk of relapse and metastasis [2]. There are many known causes of breast cancer such as family history, genetic factors, and hormones [3]. Unfortunately, the molecular targets associated with breast cancer recurrence and metastasis remain unknown. In particular, current treatment options for metastatic breast cancer are still very limited and ineffective [4]. Thus, there is a need to identify therapeutic targets and mechanisms of action associated with breast cancer metastasis.

Bone morphogenetic protein (BMP) is known to function as a tumor suppressor or an oncogene and BMP antagonists exhibit antagonism by binding directly to BMP [5]. Certain BMP antagonists such as Noggin [6] and Coco [7] have been reported to be involved in the metastasis of breast cancer cells. Gremlin-1 (GREM1) is another BMP antagonist known to be important for bone formation [8], organ development [9], and tissue differentiation [10]. GREM1 is also involved in diverse pathological conditions such as organ fibrosis [9], inflammation [11], and cancer [12,13] in BMP-dependent or -independent manners. Importantly, GREM1 is overexpressed in several types of cancers including breast cancer [12,14]. GREM1 plays an important role in the growth of breast cancer cells and is associated with poor survival of breast cancer patients [12]. In addition, GREM1 is associated with metastasis of breast cancer cells [15]. GREM1 can induce the epithelial–mesenchymal transition (EMT) process in which epithelial cells become mobile mesenchymal cells to promote cancer cell migration and tumor growth [16]. However, molecular mechanisms by which GREM1 induces breast cancer metastasis remain largely unresolved.

In cancer metastasis, EMT is required for its initiation, followed by extracellular matrix (ECM) remodeling [17]. ECM is a scaffold of extracellular macromolecules connected to cells. ECM degradation mediated by ECM-degrading enzymes such as matrix metalloproteinases (MMPs) can lead to the invasion and metastasis of malignant tumors [18]. MMPs are zinc-dependent endopetidases elevated in most cancer types. Of various MMPs, MMP13 was first identified in breast cancer and has since been reported in other types of cancer [19,20]. Tumor-derived MMP13 is associated with an aggressive tumor phenotype in breast cancer patients [21]. *MMP13* inhibition can suppress migration, invasion, and tumor formation induced by ETV4 transcription factor in mouse mammary tumorigenic cells [22]. MMP13 is also involved in metastasis of breast cancer cells induced by golgi membrane protein 1 [23]. *MMP13* knockdown markedly inhibited lung metastasis of breast cancer MCF-7 cells overexpressing *Pit-1* [24]. These findings indicate that MMP13 can increase invasive and metastatic capacities of the malignant breast cancer cells.

The objective of this study was to examine the oncogenic role of GREM1 in breast cancer. The function of GREM1 in lung metastasis of breast cancer cells was also determined. Our results showed that *GREM1* depletion inhibited tumor growth and lung metastasis *in vivo*. Notably, we found a relationship between GREM1 and MMP13 expression in breast cancer metastasis for the first time. Our results also revealed that the GREM1-MMP13 signaling pathway was triggered by activation of STAT3, suggesting that the GREM1-STAT3-MMP13 axis might be a new therapeutic target for breast cancer metastasis.

## 2. Results

### 2.1. GREM1 is a Prognostic Biomarker in Human Breast Cancer

**Figure 1:**
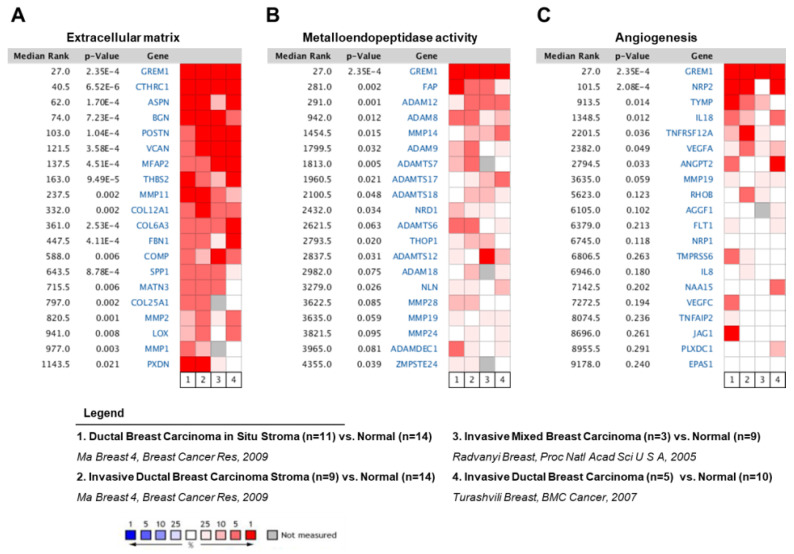
The *GREM1* gene is highly expressed in breast cancer patients. Oncomine microarray database was used to analyze *GREM1* mRNA expression in breast cancer versus normal breast tissues. The level of *GREM1* expression was compared with the levels of multiple genes corresponding to (**A**) extracellular matrix, (**B**) metalloendopeptidase activity, and (**C**) angiogenesis. Four datasets were included in the meta-analysis. Red represents overexpression and blue represents low expression. GREM1, gremlin-1.

First, we used the Oncomine database (http://www.oncomine.org) to demonstrate the role of GREM1 on the aggressive phenotype in breast cancer patients. By adding a set of genes through the extracellular matrix (Figure 1A), metalloendopeptidase activity (Figure 1B), or angiogenesis (Figure 1C) filter, the level of *GREM1* expression was compared with levels of multiple genes corresponding to each filter in four independent breast carcinoma analyses. In the meta-analysis, GREM1 expression was significantly higher in breast carcinoma tissues than in corresponding normal tissues, with a median rank of 27 and a *p*-value of 2.35 × 10^−4^. In addition, several genes associated with the extracellular matrix (Figure 1A), metalloendopeptidase activity (Figure 1B), or angiogenesis (Figure 1C) were coexpressed with GREM1 in human breast carcinomas. Results showed that *GREM1* was the most consistently highly expressed gene across multiple independent breast carcinoma analyses, indicating that GREM1 might be a potent prognostic biomarker in breast cancer patients.

### 2.2. GREM1 Contributes to the Proliferation, Migration, and Invasion of Breast Cancer Cells

**Figure 2:**
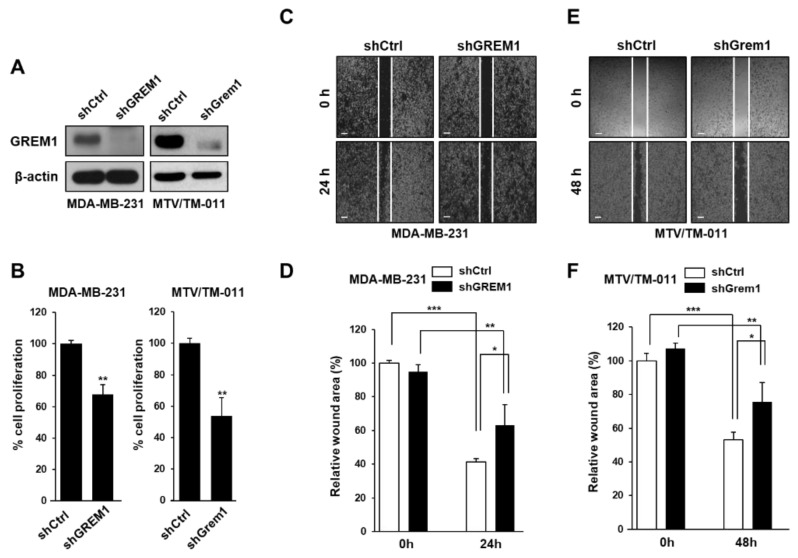
*GREM1* knockdown inhibits the growth and mobility of breast cancer cells. (**A**) Each cell line was established using a lentiviral shRNA system, and the expression level of GREM1 was evaluated by Western blot analysis. (**B**) Cells were seeded in 96-well plates and incubated for 72 h, followed by the cell proliferation assay. Two-sided *t*-test. (**C**–**F**) Cell migration was measured by using culture-inserts and the wound closure was monitored by photography at indicated time points as described in Section 4. Scale bar = 200 µm. Data are presented using triplicate wells per group and statistical significance was determined by one-way ANOVA. *, *p* < 0.05; **, *p* < 0.01; ***, *p* < 0.001.

**Figure 3:**
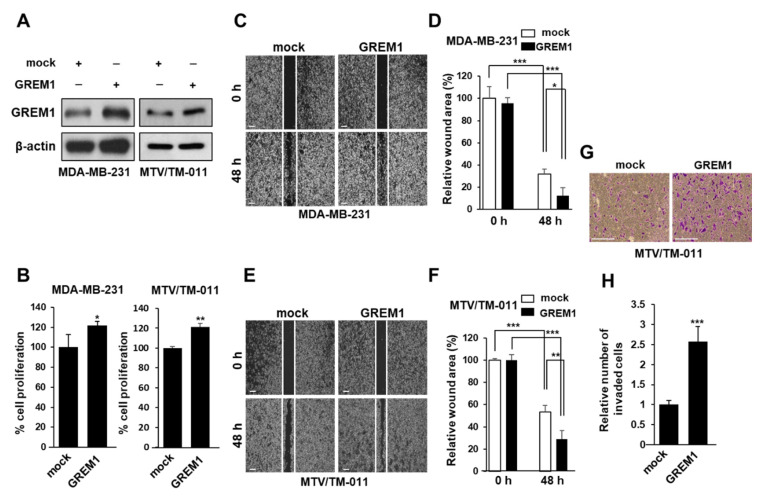
*GREM1* overexpression enhances the growth and mobility of breast cancer cells. (**A**) MDA-MB-231 and MTV/TM-011 cells were transfected with each indicated plasmid for 48 h, and the expression level of GREM1 was evaluated by Western blot analysis. (**B**) The effect of ectopic overexpression of *GREM1* on cell proliferation was examined. Two-sided *t*-test. (**C**–**F**) Cells were transiently transfected with plasmid overexpressing mock or *GREM1* for 24 h. The cells were seeded again in culture-inserts and the wound closure was monitored by photography at indicated time points as described in Section 4. (**G**,**H**) MTV/TM-011 cells were transiently transfected with plasmid overexpressing mock or *GREM1* for 24 h. The cells were seeded again in matrigel-coated inserts and incubated for 30 h, followed by the invasion assay. The invaded cells were counted and quantified using Image J. Scale bar = 200 µm. Data are presented using triplicate wells per group and statistical significance was determined by one-way ANOVA. *, *p* < 0.05; **, *p* < 0.01; ***, *p* < 0.001.

To confirm the role of GREM1 in the growth and migration of breast cancer cells, we silenced endogenous GREM1 expression in two breast cancer cell lines (MDA-MB-231 and MTV/TM-011) using a lentiviral transduction system (Figure 2A and Supplementary Figure S1A). Next, we used these cell lines to evaluate the effect of GREM1 on cell growth and noticed that *GREM1* knockdown suppressed cell proliferation (Figure 2B). To confirm the effect of GREM1 on migratory ability of breast cancer cells, we used a wound healing assay. The migration of *GREM1* knockdown cells was significantly slower compared to that of control cells (Figure 2C–F). To further elucidate the role of GREM1 in breast cancer progression, these two breast cancer cell lines were transiently transfected with a vector expressing *GREM1* (Figure 3A and Supplementary Figure S1B). *GREM1* overexpression enhanced the cell proliferation (Figure 3B) and mobility (Figure 3C–F) of these breast cancer cells. Moreover, it was confirmed that the invasive ability of cells overexpressing *GREM1* was further increased compared to the control cells (Figure 3G,H). Overall, these results indicate that GREM1 plays an important role in promoting breast cancer cell growth, migration, and invasion.

### 2.3. GREM1 is Associated with Lung Metastasis of Breast Cancer Cells

**Figure 4:**
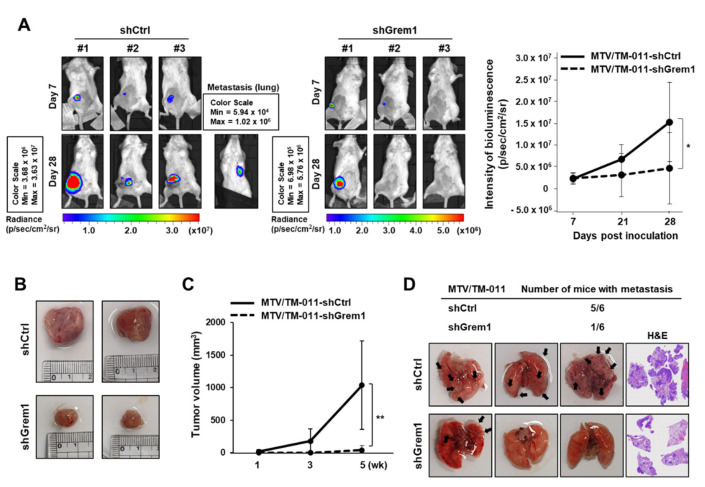
GREM1 promotes lung metastasis of breast cancer cells in vivo. (**A**) MTV/TM-011-shCtrl-luc or MTV/TM-011-shGrem1-luc cells were inoculated directly into the fourth mouse mammary fat pad of anesthetized BALB/c mice (*n* = 6/each group). Bioluminescence images of mice were captured at day 7 and day 28 after cell inoculation. (**B**) Representative images and (**C**) tumor volume of primary tumors in mice inoculated with each cell line. (**D**) Representative images of lung metastatic foci and hematoxylin and eosin staining. Black arrowheads indicate prominent lung metastatic foci. Two-sided *t*-test. *, *p* < 0.05; **, *p* < 0.01.

To investigate the role of GREM1 in breast cancer tumorigenesis and metastasis, MTV/TM-011-shCtrl-luc or MTV/TM-011-shGrem1-luc cells were orthotopically implanted into mammary fat pads of mice. Mice injected with MTV/TM-011-shGrem1-luc cells developed significantly smaller tumors than mice injected with control cells as measured by *in vivo* bioluminescence activity. On day 28 after implantation of cells into mice, the average tumor size in each group was determined by the number of photons in the region of interest (Figure 4A). The growth of tumors at the primary site showed that *Grem1* knockdown cells grew much slower than control cells, leading to a dramatic reduction in tumor volume (mean ± SD (mm^3^): 1037.812 ± 677.76 (MTV/TM-011-shCtrl-luc) vs. 41.39 ± 69.33 (MTV/TM-011-shGrem1-luc), six mice/each group) (Figure 4B,C). At about five weeks after injecting cells into mouse fat pad, the primary tumor was covered with a black cloth and bioluminescence activity was measured to confirm that the metastasized tumor grew in the lung (Figure 4A). In addition, images of lung metastasis nodules and H&E staining of entire lung tissues confirmed that mice injected with MTV/TM-011-shGrem1-luc cells had lower lung metastasis (1/6 mice) than mice injected with MTV/TM-011-shCtrl-luc cells (5/6 mice) (Figure 4D and Supplementary Figure S2). These results indicate that GREM1 is essential for the growth and metastasis of breast cancer cells.

### 2.4. GREM1 Regulates MMP13 Expression

**Figure 5:**
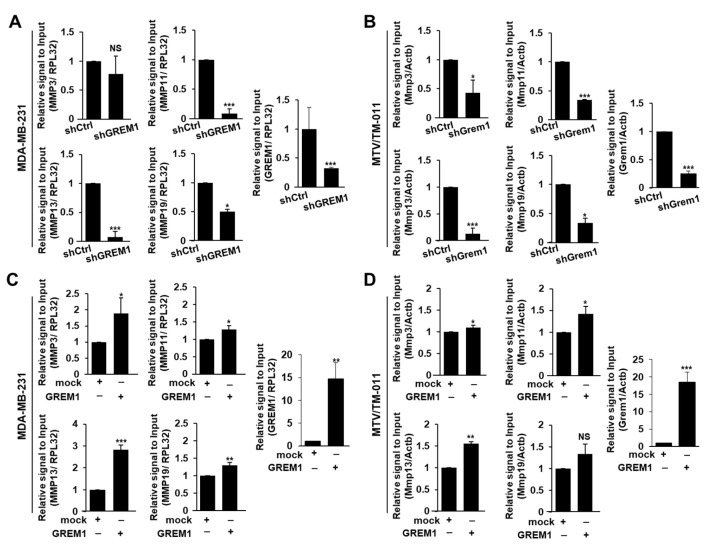
GREM1 regulates the expression of MMPs in breast cancer cells. (**A**,**B**) Relative mRNA levels of MMPs in *GREM1*-depleted breast cancer cells. (**C**,**D**) Relative mRNA levels of MMPs in *GREM1*-overexpressing breast cancer cells. MDA-MB-231 and MTV/TM-011 cells were transfected with each indicated plasmid for 48 h, and mRNA levels of genes were quantitated by qPCR analysis. Data are presented as the mean ± SD of three independent experiments. Two-sided *t*-test. *, *p* < 0.05; **, *p* < 0.01; ***, *p* < 0.001; NS, not significant.

**Figure 6:**
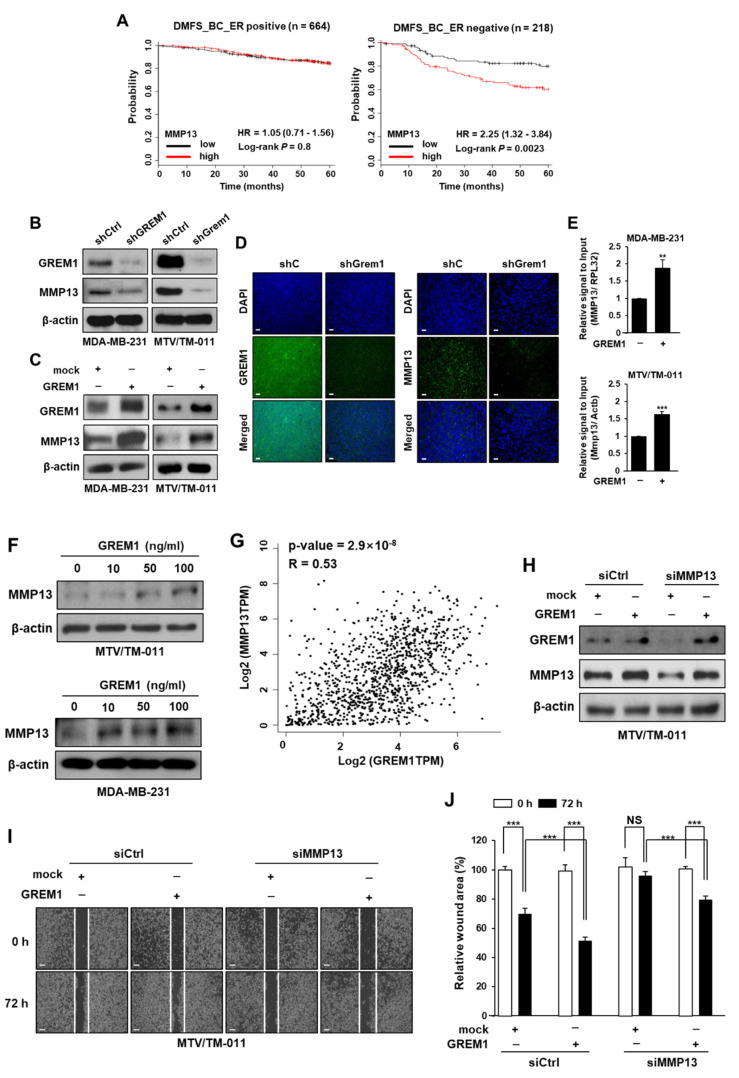
GREM1 increases the expression of MMP13. (**A**) Kaplan–Meier analysis (http://kmplot.com) of DMFS by low or high *MMP13* mRNA (*MMP13* probe set 205959_at) expression in each indicated number of breast cancer patients. DMFS, distant metastasis-free survival; HR, hazard ratio. (**B**) The effect of *GREM1* knockdown on MMP13 expression was assessed by Western blot analysis in *GREM1*-depleted cells. (**C**) Cells were transfected with each indicated plasmid for 48 h and the lysates were performed by Western blot analysis. (**D**) Representative immunofluorescence images of GREM1 and MMP13 in primary tumors formed by the injection of MTV/TM-011-luc cells expressing shCtrl or shGrem1. Scale bar = 200 μm. (**E**) Cells were treated with recombinant GREM1 protein (MDA-MB-231, 50 ng/mL; MTV/TM-011, 100 ng/mL) for 48 h, and mRNA levels of genes were quantitated by qPCR analysis. Data are presented as the mean ± SD of three independent experiments. (**F**) Cells were treated with recombinant GREM1 protein (10, 50, or 100 ng/mL) for 48 h, and the lysates were immunoblotted with the indicated antibodies. (**G**) The correlation of *GREM1* and *MMP13* mRNA in TCGA breast cancer specimens was investigated by using the web GEPIA. Correlation analysis was conducted using Spearman rank test. (**H**–**J**) MTV/TM-011 cells were transfected with siCtrl or siMMP13 (40 nM, each) for 24 h and then transfected with mock or *GREM1* for another 48 h. The lysates were subjected to immunoblot analysis (**H**). The cells were seeded again in culture-inserts and the wound closure was monitored by photography at indicated time points as described in Section 4 (**I**,**J**). Data are presented using triplicate wells per group and statistical significance was determined by one-way ANOVA. **, *p* < 0.01; ***, *p* < 0.001; NS, not significant.

To explore underlying mechanisms by which GREM1 could enhance the metastasis of breast cancer cells, we first screened representative MMPs in *GREM1*-depleted (Figure 5A,B) or *GREM1*-overexpressing (Figure 5C,D) breast cancer cells. Among MMPs, *MMP3*, *MMP11*, *MMP13*, and *MMP19* were top genes regulated by GREM1. The mRNA level of *MMP2* other than *MMP9* was also affected by GREM1 (Supplementary Figure S3). Interestingly, *GREM1* knockdown markedly inhibited MMP13 mRNA level (Figure 5A,B) whereas *GREM1* overexpression significantly enhanced MMP13 mRNA level (Figure 5C,D). We next evaluated the prognostic value of MMP13 expression in a dataset of breast cancer patients using Kaplan–Meier plotter (http://kmplot.com). Similar to *GREM1* overexpression known to be associated with worse survival of estrogen receptor (ER)-negative breast cancer patients [12], overexpression of *MMP13* was also associated with reduced distant metastasis-free survival, especially in ER-negative breast cancer patients (*MMP13* low-expression vs. high-expression patients, hazard ratio of survival: 2.25, 95% confidence interval: 1.32–3.84, *p* = 0.0023) (Figure 6A). *GREM1* knockdown inhibited protein levels of MMP13 (Figure 6B) whereas *GREM1* overexpression enhanced MMP13 protein levels in MDA-MB-231 and MTV/TM-011 cells (Figure 6C). Results confirmed that MMP13 expression was significantly decreased in tumors derived from mice injected with MTV/TM-011-shGrem1-luc cells (Figure 6D). Consistently, direct treatment with recombinant GREM1 protein increased the expression of MMP13 (Figure 6E,F). To investigate the correlation between *GREM1* and *MMP13* expression, TCGA datasets from human breast cancer tissues were obtained using the web server GEPIA [25]. As shown in Figure 6G, correlation analysis revealed a positive correlation between *GREM1* and *MMP13* (Spearman r = 0.53, *p* = 2.9 × 10^−8^). Moreover, the expression of MMP13 was suppressed in *MMP13*-knockdown cells, but restored by *GREM1* overexpression (Figure 6H). The impaired migration ability of *MMP13*-depleted cells was significantly restored by *GREM1* overexpression (Figure 6I,J). Taken together, these data suggest that GREM1 is a potent modulator of MMP13 expression.

### 2.5. GREM1 Enhances MMP13 Expression by STAT3 Activation

**Figure 7:**
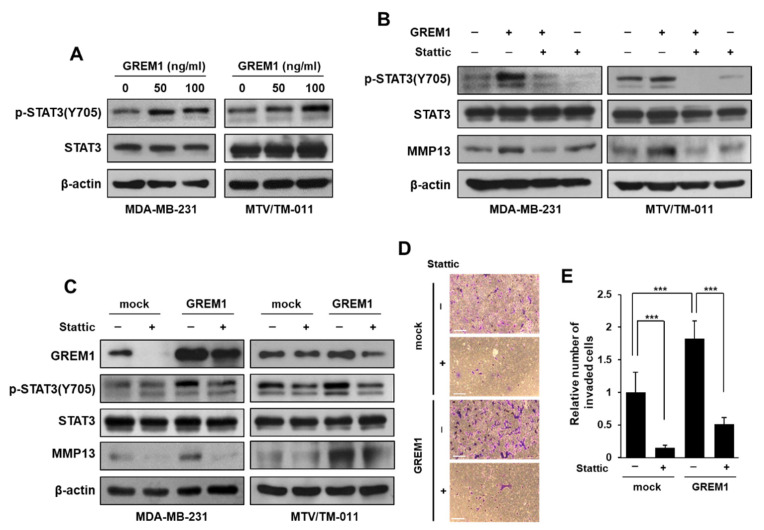
GREM1 increases MMP13 expression via STAT3 activation. (**A**) Cells were stimulated with different concentrations of recombinant GREM1 protein (10, 50, or 100 ng/mL) for 30 min, and the lysates were collected for immunoblot analysis. (**B**) Cells were incubated with Stattic (5 μM) for 2 h and then stimulated with recombinant GREM1 protein (MDA-MB-231, 50 ng/mL; MTV/TM-011, 100 ng/mL) for another 30 min (pSTAT3 and STAT3) or 48 h (MMP13). The lysates were immunoblotted with the indicated antibodies. (**C**) Cells were transfected with expression vector of mock or *GREM1* for 48 h and then incubated with Stattic (5 μM) for another 30 min (pSTAT3 and STAT3) or 24 h (GREM1 and MMP13). The lysates were subjected to immunoblot analysis. (**D**,**E**) MTV/TM-011 cells were transfected with expression vector of mock or *GREM1* for 24 h. The cells were seeded into matrigel-coated inserts with medium containing vehicle or Stattic (1 μM) and incubated for 30 h, followed by the invasion assay. Scale bar = 200 μm. Data are presented using triplicate wells per group and statistical significance was determined by one-way ANOVA. ***, *p* < 0.001.

To evaluate whether the effect of GREM1 on MMP13 expression is BMP-dependent or BMP-independent, MDA-MB-231 cells were overexpressed with each plasmid expressing mock or *GREM1*, and then treated with LDN193189, a BMP pathway inhibitor. The expression of MMP13 was significantly increased by *GREM1* overexpression and treatment of LDN193189 did not affect the increased MMP13 expression by GREM1 (Supplementary Figure S4). In our previous study, we confirmed that GREM1 promotes breast cancer proliferation through EGFR activation [12], so we confirmed that the increase in MMP13 expression by GREM1 is through EGFR activation. However, the expression of MMP13 increased by GREM1 was not reduced by the treatment of erlotinib, an inhibitor of the EGFR tyrosine kinase (Supplementary Figure S5). We then examined whether GREM1 could stimulate transcription factors involved in MMP13 expression. STAT3 is a member of the STAT protein family. It has been reported that STAT3 can induce transcription of numerous oncogenes including various MMPs [26,27]. Treatment of MDA-MB-231 and MTV/TM-011 cells with GREM1 protein induced phosphorylation of STAT3 at Tyr705 (Figure 7A). Treatment with Stattic, a STAT3 activation inhibitor, reduced MMP13 protein expression increased by GREM1 (Figure 7B). Moreover, the expression of MMP13 increased by *GREM1* overexpression was reduced in the presence of Stattic (Figure 7C). Likewise, *GREM1* overexpression increased both the migratory (Supplementary Figure S6) and invasive capacity (Figure 7D,E) of MTV/TM-011 cells, while that capability was inhibited by Stattic. Collectively, these results suggest that an increase in MMP13 expression by GREM1 occurs through STAT3 activation and that this GREM1-STAT3-MMP13 axis is important for promoting the migration and invasion of breast cancer cells.

## 3. Discussion

It is well known that excessive EMT induction causes tumor metastasis. So far, many studies have shown that overexpression of GREM1 can induce fibrosis and tumor promotion through EMT signaling. More recently, it has been reported that GREM1 is associated with breast cancer metastasis *in vivo* [15]. However, little is known about how GREM1 promotes breast cancer cell metastasis. Our present study demonstrates that GREM1 can increase tumor promotion and lung metastasis of breast cancer cells. We analyzed the effect of GREM1 on the expression of MMPs and found that MMP13 was a downstream target of GREM1. Furthermore, we investigated the effect of GREM1 on the activation of STAT3, one of representative transcription factors that can regulate MMP13 expression. Although GREM1 is known to interfere with the interaction between BMP and BMP receptors by binding to BMP [5,28], GREM1 can also act as a ligand for cell surface receptors through cysteine knot structure, one of hallmarks of signal transduction molecules [28,29]. GREM1 has been found to be able to bind directly to vascular endothelial growth factor receptor-2 [30] and epidermal growth factor receptor [12] to induce intracellular signaling. Such cellular receptor activation can increase activities of various intracellular kinases, thereby inducing the growth of cancer cells. GREM1 can also increase activities of various transcription factors involved in cancer cell proliferation or tumor promotion. GREM1 can activate Slug or Smad to induce EMT in human breast cancer cells [16] or tubular epithelial cells [31], respectively. GREM1 can promote EMT with upregulation of Snail in retinal pigment epithelial cells [32]. GREM1 can also activate nuclear factor-κB signaling, leading to subsequent induction of catabolic enzymes and osteoarthritis [33]. GREM1 can enhance the activity of estrogen-related receptor α and lead to the progression of breast cancer [12]. As such, overexpression of GREM1 can increase the activity of intracellular kinases or transcription factors that might regulate a variety of pathological conditions, particularly affecting the growth, migration, and metastasis of cancer cells.

Recently, the role of the surrounding microenvironment for tumor growth and metastasis has been emphasized [34,35,36]. It has been reported that GREM1 is overexpressed in tumor-associated stromal cells [37,38]. Interestingly, it has been suggested that GREM1 can induce cancer cell motility in the cancer invasion front which indicates the site where cancer metastasis occurs [39]. Cancer-associated fibroblasts (CAFs) are major components of tumor stroma that support cancer cells by stiffening or remodeling ECM. CAFs are known to promote invasive and metastatic phenomena of cancer cells by secreting various chemokines and cytokines [40]. GREM1 is overexpressed by CAFs of basal cell carcinomas [41]. CAFs-derived GREM1 can increase mesenchymal phenotype, stemness, and invasion of breast cancer cells [42]. GREM1 has been identified as one of enriched genes involved in stem cell niche, cell growth, and stromal–epithelial interaction in primary gastric CAFs compared to their corresponding non-CAFs [43]. Thus, GREM1 secreted from CAFs may also promote the metastasis of cancer cells.

Cancer stem cells are one of the critical cells that make up the tumor microenvironment. They can induce anticancer drug resistance and metastasis of cancer cells [44,45]. Several studies have reported that GREM1 is associated with the function of cancer stem cells. Treatment of cervical cancer cells with GREM1 can significantly increase expression levels of cancer stem cell markers and the ability of cancer cells to form spheroids [46]. Glioma cancer stem cells can secrete GREM1 to promote their stem-like features and maintenance [47]. Aberrant epithelial GREM1 can promote the persistence and/or reacquisition of stem cell properties in Lgr5-negative progenitor cells to form ectopic crypts, proliferate, accumulate somatic mutations, and initiate colonic tumorigenesis [13].

Since BMPs have dual functions in cancer [48], whether the mechanism by which GREM1 promotes cancer is related to BMPs remains unclear. According to studies on carcinogenic function of BMPs, inhibition of BMP signaling by inhibitor of BMP receptor DMH1 [49] or LDN-193189 [50] can suppress tumor promotion in vivo. In contrast, many studies have also reported that BMPs have function as tumor suppressors. Overexpression of BMP6 or BMP7 is associated with higher infiltration of anticancer immune cells and better prognosis in ER-positive breast cancer [51]. Furthermore, Coco, a BMP inhibitor, can induce and reactivate breast cancer cells at lung metastatic sites by blocking BMP signaling [7]. As such, BMPs and BMP antagonists might have specific effects on cancer cells as a result of their interactions. However, GREM1 has been reported to function independently of BMPs in the proliferation, migration, and invasion of cancer cells [52]. In particular, unlike GREM1, there is no correlation between high BMP mRNA expression and poor prognosis in ER-negative breast cancer patients [15]. Therefore, it is necessary to evaluate the function and mechanism of GREM1 by focusing on the molecular exchange between cells in terms of the tumor microenvironment rather than the correlation between GREM1 and BMP in cancer cells themselves.

Although many studies have reported the tumor promoting function of GREM1, mechanisms involved in the overexpression of GREM1 and mechanisms by which GREM1 is involved in the growth of cancer cells remain unclear. Several microRNAs such as microRNA-27a/b [53,54], -137 [55], and -23b/27b cluster [56] can inhibit the expression of GREM1. However, inhibitors targeting GREM1 have not been developed yet. To develop GREM1-specific inhibitors and drugs, further studies are needed to clarify mechanisms regarding the function of GREM1 in tumors. We, for the first time, showed that GREM1 could regulate multiple MMPs, especially MMP13, in breast cancer cells. GREM1 could activate STAT3 which is involved in the expression of MMPs. In this context, the GREM1-STAT3-MMP13 axis can be a promising therapeutic target for treating cancers, especially lung metastasis of breast cancer cells.

## 4. Materials and Methods

### 4.1. Cell Culture and Reagents

MDA-MB-231 and MTV/TM-011 cells were originally obtained from American Type Culture Collection and Korean Cell Line Bank, respectively. MDA-MB-231 cells were cultured in DMEM (Corning Inc., Corning, NY, USA) containing 10% fetal bovine serum (FBS, Thermo Fisher Scientific, Waltham, MA, USA) and 1% penicillin/streptomycin (Corning Inc.). MTV/TM-011 cells were cultured in RPMI (Corning Inc.) containing 10% FBS and 1% penicillin/streptomycin. Cells were maintained at 37 °C in a humidified atmosphere with 5% CO_2_/95% air. Rabbit polyclonal GREM1 antibody (Cat#. 140010) was purchased from Abcam (Cambridge, UK). Recombinant human GREM1 (Cat#. 5190-GR) was obtained from R&D systems (Minneapolis, MN, USA). Anti-MMP13 antibody (Cat#. 515284) was purchased from Santa Cruz Biotechnology (Dallas, TX, USA). Anti-phospho STAT3 (Cat#. 9145s) and anti-STAT3 (Cat#. 9139s) were obtained from Cell Signaling Technology (Danvers, MA, USA). Anti-β-actin antibody (Cat#. A1978) was purchased from Sigma-Aldrich (St. Louis, MO, USA). Stattic (CAS. No. 2798) was purchased from Tocris (Bristol, UK).

### 4.2. Gene Silencing

Human TRC lentiviral GREM1 shRNA (TRCN0000063837), mouse TRC lentiviral Grem1 shRNA (TRCN0000098304), and shControl (shCtrl, SHC002) were obtained from Dharmacon (Lafayette, CO, USA). Lentiviruses were packaged in 293T cells. The cells were transiently transfected with shRNA vector together with pCMV-VSV-G and pCMV-dR8.91 using Lipofectamine^TM^ 2000 reagent (Invitrogen, Carlsbad, CA, USA Cat#. 11668019). After transfection for 72 h, the viral supernatant was collected, filtered, and used for transduction of breast cancer cells in the presence of 8 μg/mL polybrene (Merck Millipore, Burlington, MA, USA Cat#. TR-1003-G). Stable cell lines were selected with 1 μg/mL puromycin (InvivoGen, San Diego, CA, USA Cat#. ant-pr-1) for a week and confirmed by Western blot or qPCR analysis. Endogenous MMP13 was knocked down using specific small interfering RNA (siRNA) (Bioneer, Daejeon, Korea, Cat#. 17386). Briefly, cells were transiently transfected with siRNAs by reverse transfection using Lipofectamine RNAiMAX (Invitrogen).

### 4.3. Reverse Transcription-Quantitative Polymerase Chain Reaction (RT-qPCR)

Total RNA was isolated from cells using TRIzol^®^ (Invitrogen, Cat#. 15596026). Reverse transcription of total RNA was performed using M-MLV reverse transcriptase (Promega, Madison, WI, USA Cat#. M1705) according to the manufacturer’s instructions. Quantitative PCR (qPCR) was performed using TOP real^TM^ qPCR 2X Pre-MIX (Enzynomics, Daejeon, Korea, Cat#. RT501S) and StepOnePlus Real-Time PCR (Thermo Fisher Scientific). Primer sequences are listed in the Supplementary Table S1.

### 4.4. Western Blot Analysis

Standard sodium dodecyl sulfate-polyacrylamide gel electrophoresis (SDS-PAGE) and Western blotting were used to analyze the expression of various proteins. Cell lysates were prepared using lysis buffer (Cell Signaling Technology, Cat#. 9803) containing protease inhibitors and phosphatase inhibitors (Roche, Basel, Switzerland). The quantitative protein concentration was determined by BCA Protein Assay Kit (Thermo Fisher Scientific) and equal amounts of protein were loaded on 8–12% SDS-PAGE. Proteins were transferred to polyvinylidene difluoride membrane (Merck Millipore) and subjected to immunoblotting using various antibodies overnight at 4 °C, followed by further incubation with the secondary antibody (AbFrontier, Seoul, Korea, Cat#. LF-SA8001 and LF-SA8002) at room temperature for 1 h. Visualization of protein bands was detected with Westsave Gold detection reagents (AbFrontier, Cat#. LF-QC0103).

### 4.5. Cell Proliferation Assay

Cells were seeded in 96-well plates (1 × 10^4^/well) and incubated for 3 days. The cells were then treated with 20 μL of CellTiter 96^®^ Cell Proliferation Assay (Promega, Cat#. G3582) for 2 h at 37 °C. The absorbance of each well was detected at 490 nm with a Multiskan GO microplate reader (Thermo Fisher Scientific). All procedures were performed according to the manufacturer’s instructions.

### 4.6. Wound Healing Assay

MDA-MB-231 and MTV/TM-011 cells were seeded at the density of 3 × 10^5^ mL into Ibidi Culture Inserts (Ibidi, Gewerbehof, Germany). After incubation for 24 h, Ibidi Culture Inserts were gently removed. Cells were incubated at 37 °C for an additional 24 or 48 h. Images of wound sites were captured at 0 h (control) and indicated time points using an inverted microscope (4× magnification). Each wound area was determined using Image J software.

### 4.7. Invasion Assay

MDA-MB-231 and MTV/TM-011 cells (1 × 10^5^ cells) were suspended in serum-free medium containing DMSO or Stattic and seeded into the upper transwell inserts (Corning Inc., Cat#. 354480). The lower chambers were filled with medium containing 20% FBS. After incubation for 30 h, the bottom of transwell inserts was fixed with cold methanol and stained with 0.5% crystal violet. The non-invaded cells were wiped off and the invaded cells were counted in four randomly selected fields using an inverted microscope (100× magnification).

### 4.8. Mouse Orthotopic Model

All animal experiments were conducted on protocols approved by the Institutional Animal Care and Use Committee of the Soonchunhyang University (SCH19-0033). Female BALB/c mice, 5 weeks of age (weight 18–20 ± 1–2 g) were purchased from Orient Bio Inc. (Seongnam, Korea). Mice were controlled in specific pathogen free conditions: 20–24 °C, 12/12 h of dark/light cycle, 60 ± 5% humidity, and plastic cage (four mice/cage). For the orthotopic breast cancer mouse model, MTV/TM-011-shCtrl-luc or MTV/TM-011-shGrem1-luc cells (1 × 10^5^ cells, each) were suspended in 60 μL of HBSS and inoculated into fourth mouse mammary fat pads after isoflurane inhalation anesthesia. Both the volume of the primary tumors and the body weight of mice were measured twice a week. Tumor growth was monitored via bioluminescence imaging (IVIS Lumina, PerkinElmer, Waltham, MA, USA) available at the Soonchunhyang Biomedical Research Core-facility of Korea Basic Science Institute (KBSI). At the end of the experiments, mice were euthanized by CO_2_ inhalation and each tumor was removed.

### 4.9. Immunofluorescence Staining

For staining fixed paraffin-embeded tissues, a standard protocol for deparaffinization, antigen retrieval, and permeabilization was followed. The tissues were incubated overnight at 4 °C with primary antibodies. The tissues were washed with phosphate-buffered saline and incubated with Alexa Fluor^®^ 488 antibody (Invitrogen, Cat#. A-11094) for 1 h at room temperature. After washing, the tissues were mounted by the ProLong^®^ Gold Antifade Mountant with DAPI (Invitrogen, Cat#. P-36935) and examined under the fluorescence microscope.

### 4.10. Bioinformatics Meta-Analysis

To investigate the expression profile of GREM1 in human breast cancer versus normal breast tissue, we conducted a meta-analysis in the Oncomine database (http://www.oncomine.org) by setting the following search items: “GREM1”, “extracellular matrix”, “metalloendopeptidase activity”, “angiogenesis”, and “Breast Cancer vs. Normal Analysis”. All procedures were performed according to the instructions provided by Oncomine.

### 4.11. Statistical Analysis

Data were expressed as the mean ± SD of results obtained from at least three independent experiments. Significant differences were determined by a Student’s *t*-test or one-way ANOVA. A *p*-value of less than 0.05 was considered to be statistically significant. *, *p* < 0.05; **, *p* < 0.01; and ***, *p* < 0.001.

## Supplementary Materials

Supplementary Materials can be found at https://www.mdpi.com/1422-0067/21/23/9227/s1.
