# Supplementary material for: Gremlin-1 Promotes Metastasis of Breast Cancer Cells by Activating STAT3-MMP13 Signaling Pathway
Source: Int J Mol Sci. 2020 Dec 3;21(23):9227. doi: 10.3390/ijms21239227 (PMC7730512; doi:10.3390/ijms21239227)
Supplement: Supplementary file 1 [file ijms-21-09227-s001.pdf]

Supplementary Figure Legends

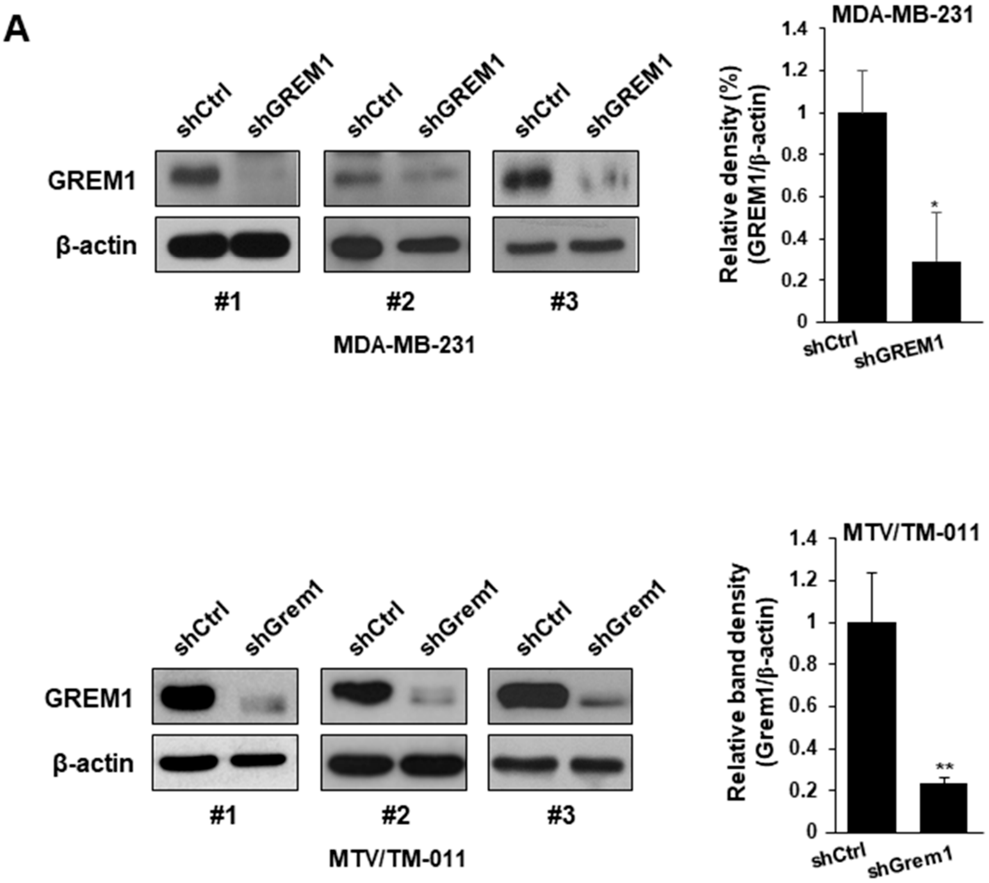

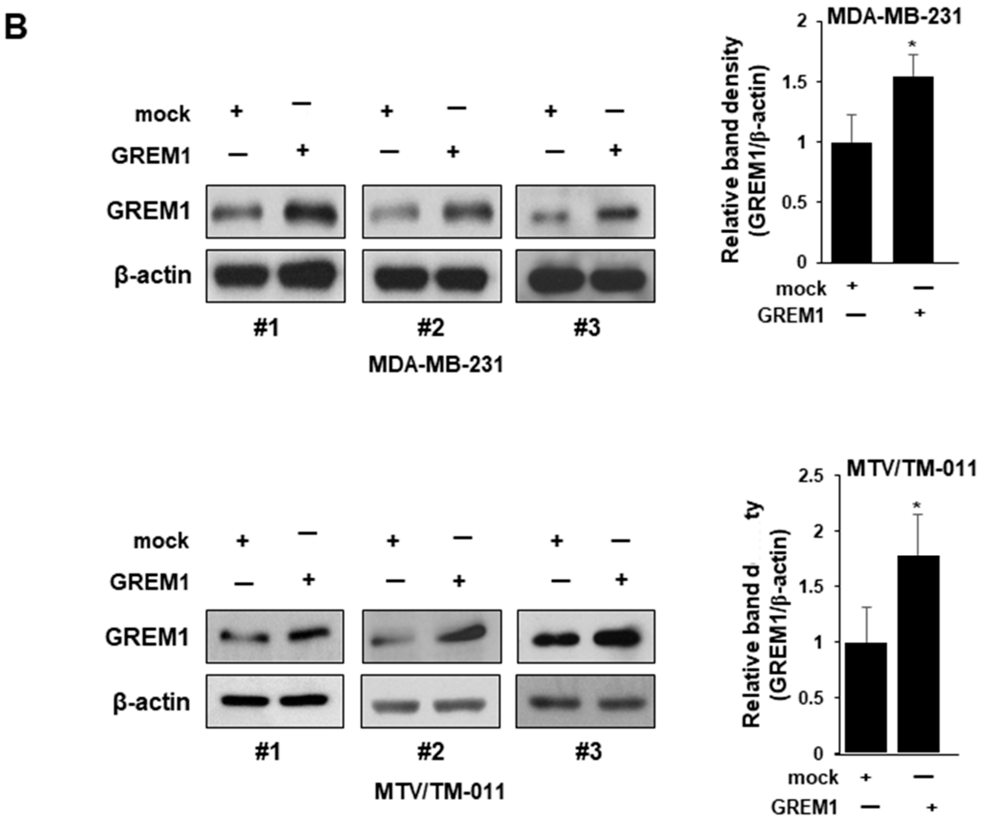

Supplementary Fig. 1. The expression of GREM1 was verified in each indicated cell line. (A) The cell lysates of MDA-MB-231 and MTV/TM-011 cell lines stably expressing shCtrl or shGREM1 were immunoblotted with GREM1 antibody. **(B)** MDA-MB-231 and MTV/TM-011 cells were transfected with each plasmid expressing mock or GREM1 for 48 h and the lysates were immunoblotted with GREM1 antibody. All quantitative values were obtained using three sets of independently separated samples on different days. Data are presented as the mean ± SD of three independent experiments. Two-sided t test. *, $p < 0.05$; **, $p < 0.01$.

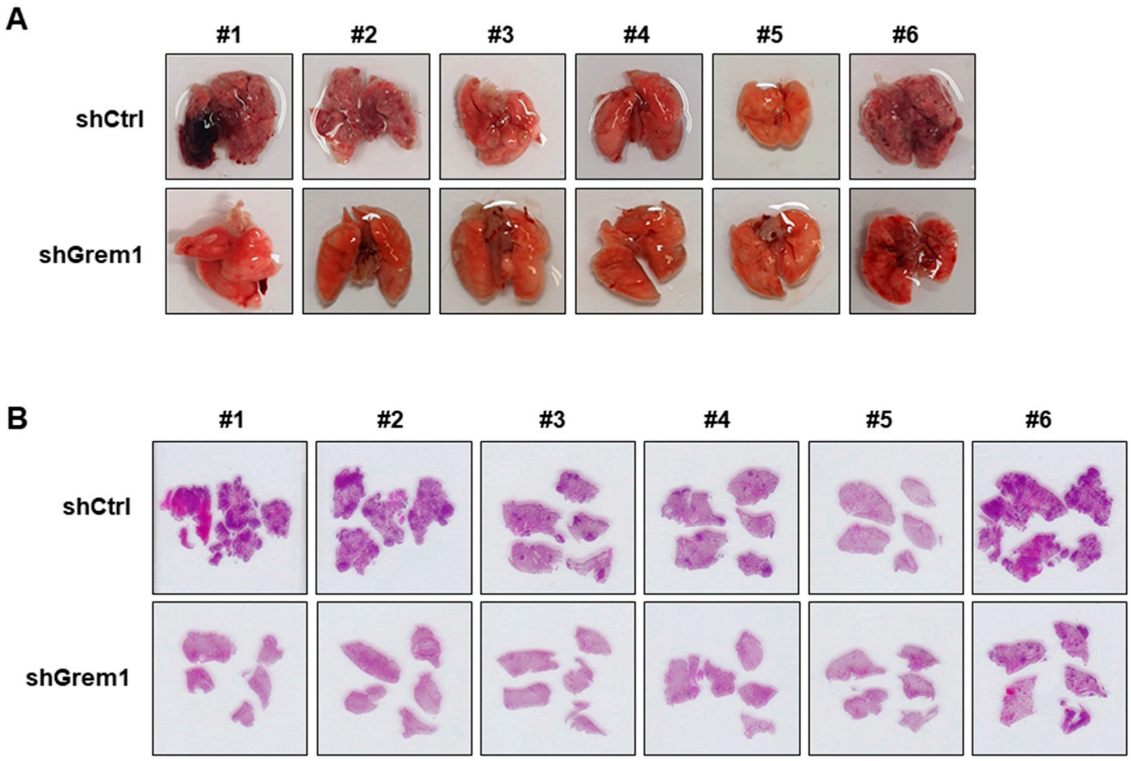

Supplementary Fig. 2. GREM1 knockdown suppressed lung metastasis of breast cancer cells. (A) Images of lung metastasis nodules formed by the injection of MTV/TM-011 cells expressing shCtrl or shGrem1 into the mouse mammary fat pads (n = 6/ each group). **(B)** Whole lung tissues were stained by H&E (n = 6/ each group).

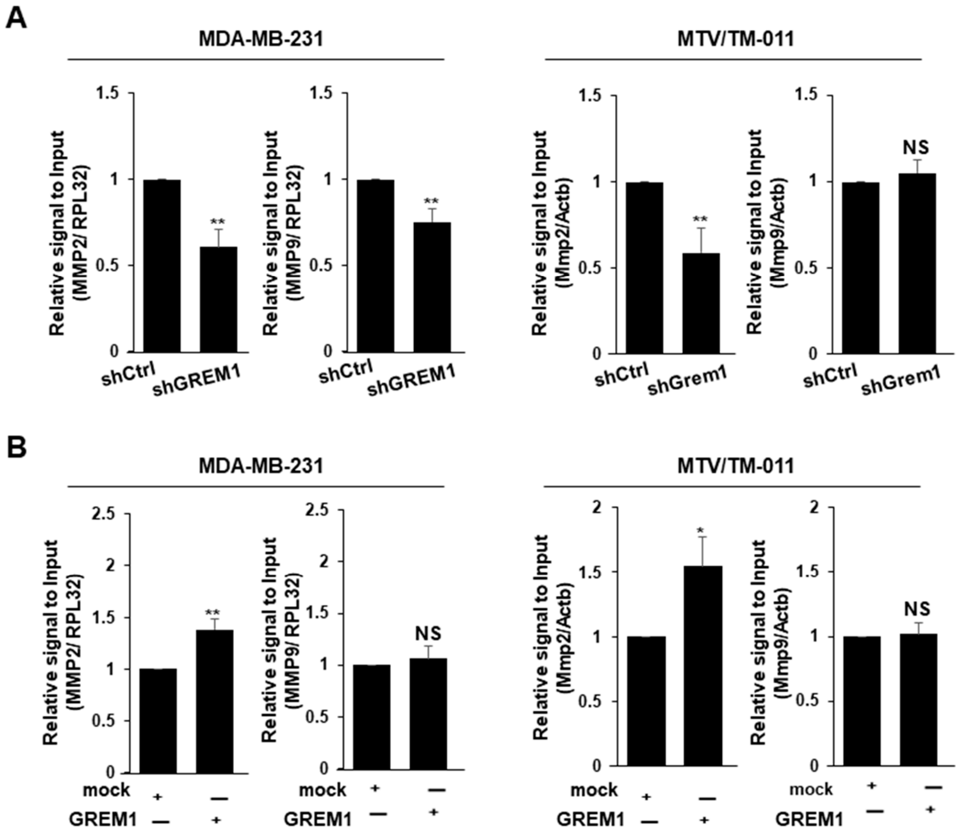

Supplementary Fig. 3. GREM1 regulated the expression of MMP2 or MMP9. (A) Relative mRNA levels of *MMP2* and *MMP9* in GREM1-depleted breast cancer cells. **(B)** Relative mRNA levels of *MMP2* and *MMP9* in GREM1-overexpressing breast cancer cells. MDA-MB-231 and MTV/TM-011 cells were transfected with each indicated plasmid for 48 h, and mRNA levels of genes were quantitated by qPCR analysis. Data are presented as the mean ± SD of three independent experiments. Two-sided t test. *, $p < 0.05$; **, $p < 0.01$; NS, not significant.

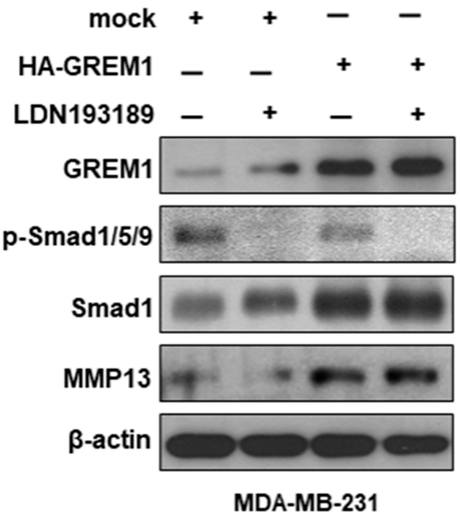

Supplementary Fig. 4. The expression of MMP13, which was increased by GREM1, was not affected by treatment of LDN193189, a BMP pathway inhibitor. MDA-MB-231 cells were transfected with each plasmid expressing mock or GREM1 for 24 h and then incubated in the absence or presence of LDN193189 (1 μM) for another 24 h. The protein lysates were immunoblotted with each indicated antibody.

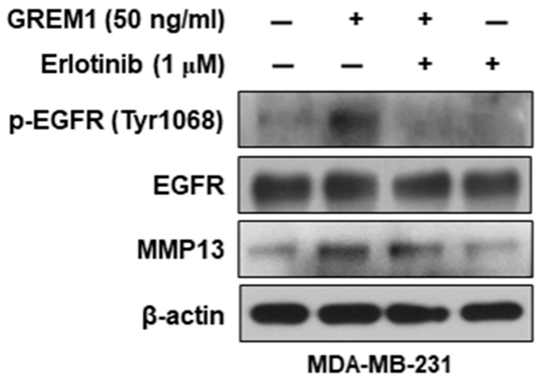

Supplementary Fig. 5. The expression of MMP13, which was increased by GREM1, was not reduced by treatment of erlotinib, an EGFR tyrosine kinase inhibitor. MDA-MB-231 cells were pretreated with erlotinib (1 μM) for 24 h and then incubated with GREM1 (50 ng/ml) for another 30 min (pEGFR and EGFR) or 48 h (MMP13). The protein lysates were subjected to immunoblot analysis.

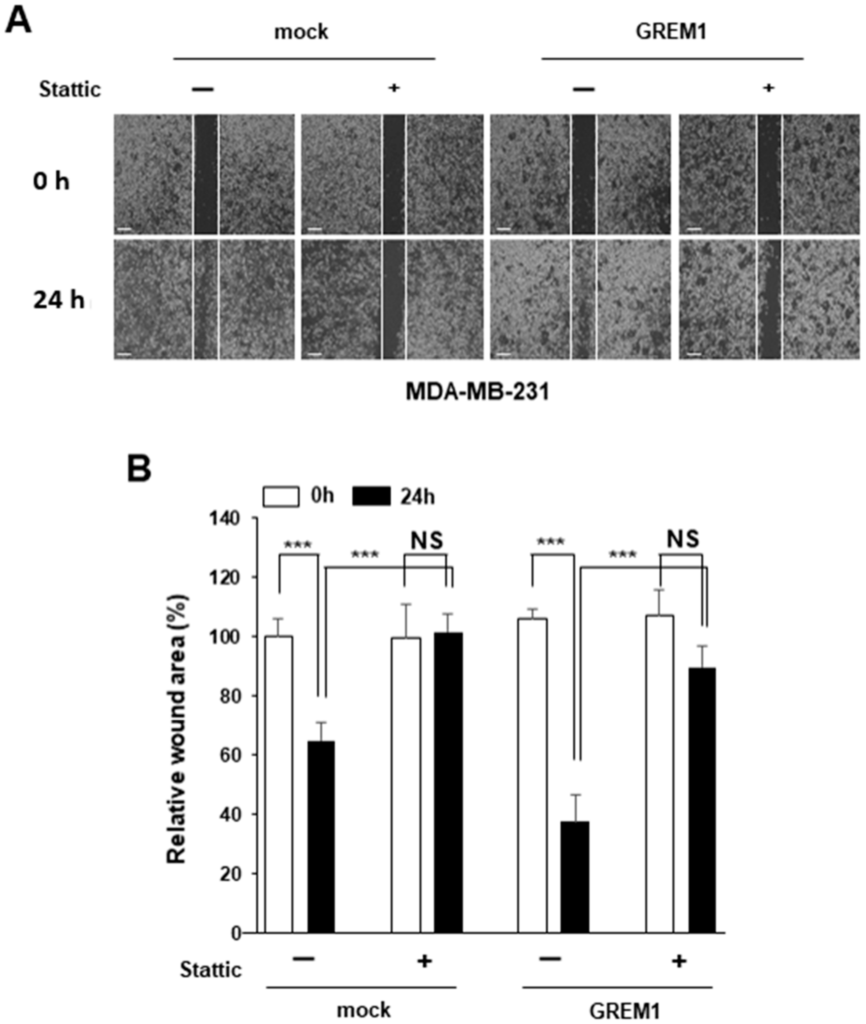

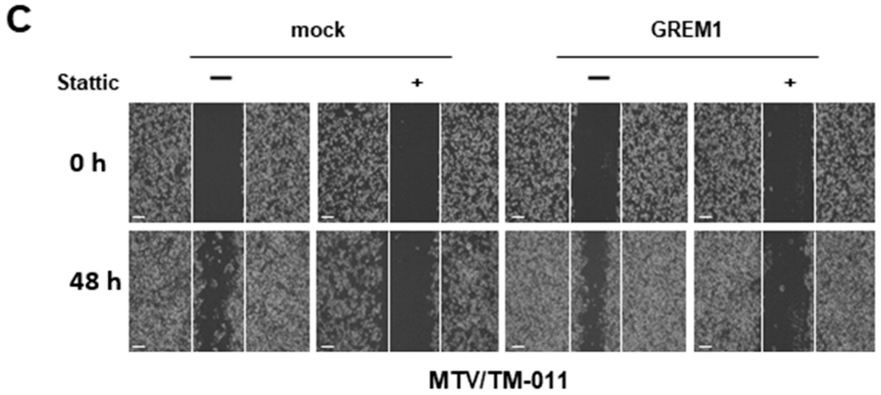

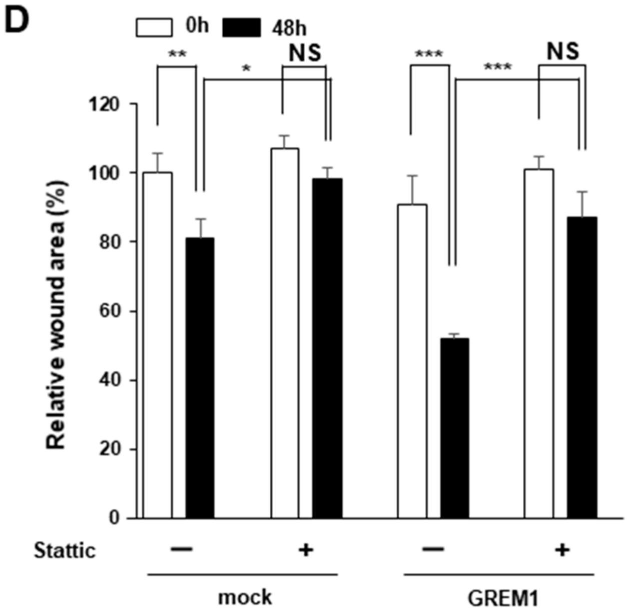

Supplementary Fig. 6. The enhanced migratory capacity of GREM1-overexpressing cells was inhibited by Stattic treatment. (A-D) MDA-MB-231 and MTV/TM-011 cells were transiently transfected with plasmid ovexpressing mock or GREM1 for 48 h and seeded again in culture-inserts with medium containing Stattic (5 μM). Images of wound sites were captured at 0 h (control) and the indicated time periods of incubation using an inverted microscope (4 x magnification). Scale bar = 200 μm. Each wound area was determined using Image J software. Data are presented using triplicate wells per group and statistical significance was determined by one-way ANOVA. *, $p < 0.05$; **, $p < 0.01$; ***, $p < 0.001$; NS, not significant.

Supplementary Table 1. qRT-PCR primer sequences

Primers (Human)	Forward	Reverse
GREM1	TCATCAACCGCTTCTGTTACG	GGCTGTAGTTCAGGGCAGTT
MMP2	GATACCCCTTTGACGGTAAGGA	CCTTCTCCCAAGGTCCATAGC
MMP3	CGGTTCCGCCTGTCTCAAG	CGCCAAAAGTGCCTGTCTT
MMP9	AGACCTGGGCAGATTCCAAAC	CGGCAAGTCTTCCGAGTAGT
MMP11	GGGTGTACGACGGTGAAAAG	GTGGAAACGCCAGTAGTCCC
MMP13	CCAGACTTCACGATGGCATTG	GGCATCTCCTCCATAATTTGGC
MMP19	GCAATGTGGCTCCCTTGAC	TCAGTCCAGAACTCGTCTTCG
RPL32	TTAAGCGTAACTGGCGGAAAC	AAACATTGTGAGCGATCTCGG

Primers (Mouse)	Forward	Reverse
Grem1	GGGACCCTACTGCCAACAG	TTTGCACCAATCTCGCTTCAG
Mmp2	ACCTGAACACTTTCTATGGCTG	CTTCCGCATGGTCTCGATG
Mmp3	TTAAAGACAGGCACTTTTGGCG	CCCTCGTATAGCCCAGAACT
Mmp9	CTGGACAGCCAGACACTAAAG	CTCGCGGCAAGTCTTCAGAG
Mmp11	CCGGAGAGTCACCGTCATC	GCAGGACTAGGGACCCAATG
Mmp13	CTTCTTCTTGTTGAGCTGGACTC	CTGTGGAGGTCACTGTAGACT
Mmp19	CTGTGGCTGGCATTCTTACTT	GGGCAGTCCAGATGCTTCC
Actb	GGCTGTATTCCCCTCCATCG	CCAGTTGGTAACAATGCCATGT
